# The clinical features and real world treatment outcomes in von Hippel Lindau related retinal capillary hemangioblastomas

**DOI:** 10.1186/s40942-025-00721-1

**Published:** 2025-08-18

**Authors:** Abdulrahman Khan, Wael A. Alsakran, Abdullah Barry, Abdulrahman H. Badawi, Moustafa S. Magliyah

**Affiliations:** 1 Vitreoretinal Division, King Khaled Eye Specialist Hospital, Riyadh, Saudi Arabia; 2 Vitreoretinal and Uveitis Division, King Khaled Eye Specialist Hospital, PO Box 7141, Riyadh, 11462 Saudi Arabia

**Keywords:** Von Hippel-Lindau, Retinal capillary hemangioblastoma, Exudative retinal detachment, Tractional retinal detachment, Pars plana vitrectomy

## Abstract

**Background and objective:**

To present the clinical features and outcomes of treatment in von Hippel-Lindau (VHL) related retinal capillary hemangioblastoma (RCH).

**Methods:**

A retrospective interventional cohort study that included Patients who were diagnosed with VHL-related RCH from 2014 to 2024 at a tertiary referral center.

**Results:**

Thirty-two eyes of 18 patients with VHL-associated RCH were included. Treatment was indicated in 30 eyes (90.9%), and the indications for treatment were tumor leakage and subretinal fluids (SRF) with or without exudative retinal detachments in 25 eyes (83.3%) and tractional/exudative retinal detachments (T-ERD) in 4 eyes (13.3%). Significantly worse visual outcomes were observed in patients who had lower BCVA on presentation, larger tumor sizes, developed retinal complications, underwent retinal surgery, detached retina on the last follow up and had persistent tumor outcomes.

**Conclusion:**

The management outcomes in VHL-related RCH depend on the lesionsize and location, severity of the clinical picture on presentation and the development of complications.

**Clinical trial number:**

Not applicable.

**Supplementary Information:**

The online version contains supplementary material available at 10.1186/s40942-025-00721-1.

## Introduction

Von Hippel-Lindau (VHL) disease is a multisystemic neoplastic disease that involves multiple organs and body systems, and the typical ophthalmic manifestation of the VHL disease is retinal capillary hemangioma (RCH) [1]. RCH was first described by Eugen von Hippel as angiomatosis retinae [2]. The Swedish pathologist, Arvid Lindau, described the association between cerebellar and RCH [3].

Heterozygous variants in the *VHL* gene, which is located on the short arm of chromosome 3, at locus 3p25-3p26, were found to lead to the manifestation of VHL disease [4]. The *VHL* gene is a tumor suppressor gene that consists of three coding exons and encodes the VHL protein [5]. Inactivation of the VHL protein leads to the activation of several angiogenic factors, including platelet-derived growth factor, vascular endothelial growth factor (VEGF), erythropoietin, plasminogen activator inhibitor-1, and transforming growth factor a. These factors contribute to increased angiogenesis and neoplastic formation in patients with VHL [6].

RCH is a prominent manifestation of VHL disease, which is found in 49–68% of patients with VHL disease can lead to various complications, including macular exudation and edema, epiretinal membranes (ERM), vitreous hemorrhage (VH), exudative retinal detachments (ERD) and tractional retinal detachment (TRD) [7–10].

Despite the recent advances in treatment modalities, the rate of blindness in VHL-related RCH was reported to be about 8.2% [11, 12]. In this study, we describe the clinical features and outcomes of multiple treatment modalities in VHL-related RCH, and report the long-term anatomical and visual outcomes.

## Methods

This was a retrospective chart review that included all patients with VHL disease at King Khaled Eye Specialist Hospital (KKESH) between January 2007 and December 2024. The study was approved by the Institutional Review Board (IRB) at KKESH (IRB number: RP 25078-R). This study adhered to the tenets of the Declaration of Helsinki. The patients were included if they were diagnosed with VHL disease and had unilateral or bilateral RCH, with a minimum follow-up of one year. The collected data included patient demographics, best corrected visual acuity (BCVA) at presentation and last visit, intraocular pressure (IOP), ophthalmic examination details, treatment modalities, the response and effects of treatments, extraocular manifestations, ocular complications, and surgical interventions. Details of the RCH included lesion size (in disc diameters), number of lesions, location of lesions and retinal and ophthalmic complications. All treatment modalities for RCH and their outcomes were recorded, including focal laser, intravitreal injections and surgical procedures. The focal laser application approach was using long durations, large spot sizes and low to medium power, and the laser was applied directly to the lesions. The definition of blindness among the study eyes was a final vision of light perception (LP) or no light perception (NLP).

### Statistical analysis

A descriptive analysis was performed, and categorical variables were reported as frequencies and percentages, while continuous variables were reported as mean (SD), range [min–max]. The visual outcomes were considered worse visual outcomes if they were less than 20/200. Statistical analysis was performed using the multivariate binary logistic regression analysis to investigate factors that were associated with worse visual outcomes, and for treatments which were associated with significant visual improvements. The confidence interval was set to 95% where a corresponding *P* value less than 0.05 was considered statistically significant. Table 1

## Results

### Patient demographics

Thirty-two eyes of 18 patients with VHL-associated RCH were included. 12 patients (66.7%) were males and 7 were females (33.3%). The mean age was 34.21 ± 13.6 years. The mean age of presentation was 21.6 ± 8.3 years. The mean duration of follow-up was 11.03 ± 8.07 years. Nine patients (50%) had a positive family history of VHL disease. Six patients (33.3%) had cerebellar hemangiomas, 6 patients (33.3%) had kidney tumors, 5 patients (27.8%) had pancreatic cysts, 3 patients (16.7%) had pheochromocytomas, while 4 patients (22.2%) had no systemic associations. One patient had pulmonary embolism, and one patient had a concomitant enhanced S cone syndrome, which was proven by whole exome sequencing (WES). Fourteen patients (73.7%) had bilateral disease. Seven (36.8%) patients had WES. The results of WES are summarized in Supplementary Table 1. All tested patients had heterozygous variants in the *VHL* gene, while one patient had a concomitant homozygous variant in the *NR2E3* gene.

### Clinical findings

The mean best-corrected visual acuity (BCVA) on presentation was 0.69 ± 0.9 (Snellen = 20/100). The mean IOP was 16.2 ± 5 mmHg. The mean number of lesions was 3 ± 2.8 lesions. Juxta-papillary RCH was found in three eyes (9.1%), and juxtafoveal RCHs were found in 3 eyes (9.1%). The mean tumor size in disc diameters was 1.5 ± 0.9 disc diameters (DD). The mean number of lesions found in the superotemporal quadrant was 1.12 ± 1.3 lesions, while 0.94 ± 1.2 lesions were found in the inferotemporal quadrant, 0.55 ± 0.9 lesions were found in the supero-nasal quadrant, and 0.3 ± 0.64 lesions were found in the inferonasal quadrant.

### Treatment and outcomes

**Fig. 1:**
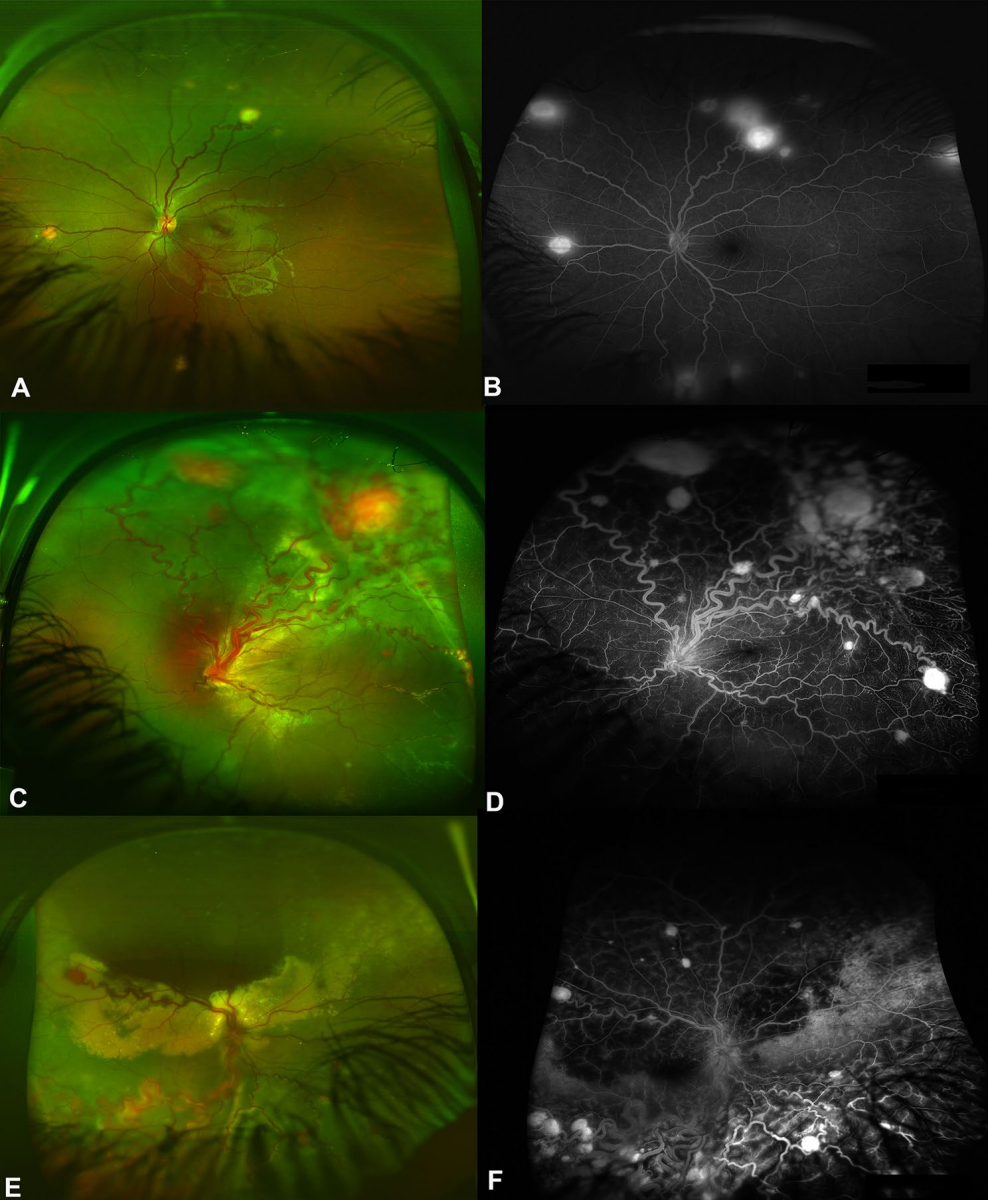
Different presentations of active retinal capillary hemangioblastoma (RCH) lesions in von Hippel-Lindau disease patients. Figure 1 is a color fundus photo of the left eye showing multiple RCH in the midperiphery of the inferior, nasal and temporal retina. B is a fundus fluorescein angiography (FFA) of the left eye showing fluorescein leakage from the RCH lesions as well as two additional areas supero-temporally and supero-nasally, indicating active RCH. B is a color fundus photo of the left eye showing multiple RCH lesions occupying the superior hemiretina with prominent feeder vessels for each tumor as well as the accumulation of subretinal fluids (SRF) in the superior retina. D is an FFA showing the leakage of the multiple RCH lesions as well as the hyper-fluorescence of the prominent feeder blood vessels. E is a color fundus photo of the right eye showing multiple RCH lesions in all retinal quadrants with macular-involving exudative retinal detachment and prominent retinal exudation. F is an FFA of the right eye showing the leakage of the multiple RCH lesions as well as the hyper-fluorescence of the prominent feeder blood vessels. Note the blockage-induced hypofluorescence in the areas of extensive retinal exudation

**Fig. 2:**
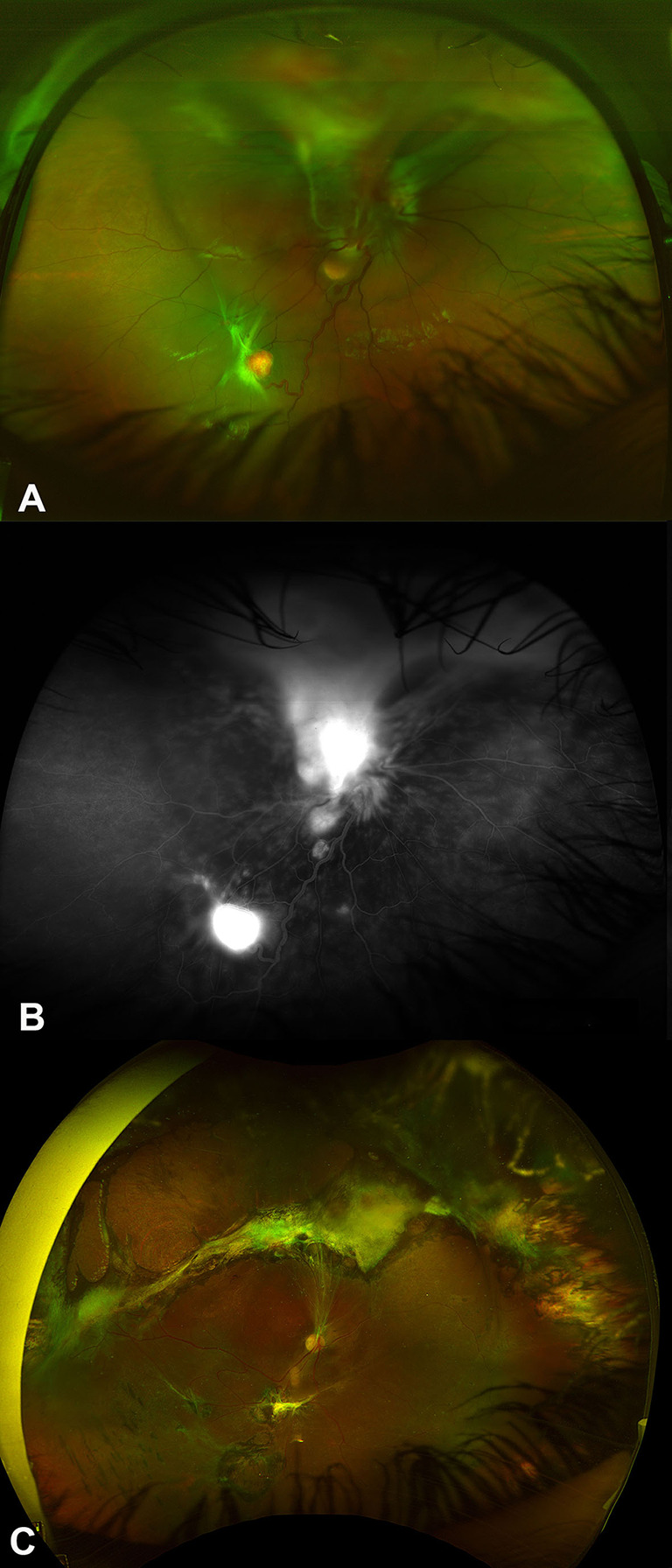
The combined tractional and exudative retinal detachment (TERD) presentation in retinal capillary hemangioblastoma (RCH) associated with von Hippel-Lindau (VHL) disease. A is a color fundus photo showing a superior RCH with adjacent subretinal fluids (SRF) and a tractional membrane extending to the macular area, causing tractional retinal detachment (TRD) along with the exudative component, as well as an inferotemporal RCH with SRF and a tractional band around it. In addition, a juxta-papillary RCH. B is a fundus fluorescein angiography (FFA) showing leakage from the superotemporal and inferotemporal RCH with subretinal accumulation of the dye superiorly and mild hypofluorescence corresponding to the juxta-papillary RCH. C is a color fundus photo of the right eye after pars plana vitrectomy and tumor excision with adjacent retinectomy and laser photocoagulation showing regression of the RCH

Treatment was indicated in 30 eyes (90.9%) and the indications for treatment were tumor leakage and subretinal fluids (SRF) with or without exudative retinal detachments (Fig. 1A-F) in 25 eyes (83.3%), and tractional/exudative retinal detachments (T-ERD) (Fig. 2A-C) in 4 eyes (13.3%) and choroidal neovascular membrane (CNVM) in one eye of a patient who was diagnosed with VHL and enhanced s-cone syndrome (ESCS). Table 2 summarizes the treatment modalities used in RCHs. The mean number of laser sessions was 1.96 ± 1.3 sessions. Ten out of 27 eyes (37%) treated with laser photocoagulation alone achieved long-term tumor regression. The mean number of injections was 2.3 ± 1.4 injections. Thirteen eyes required retinal surgical interventions. Two eyes had scleral drainage surgeries and were followed by vitrectomy in one eye, while 12 eyes required pars plana vitrectomy. The mean number of vitrectomies was 2 ± 1.0 surgeries. The indications for retinal surgical interventions included and included persistent ERD in 7 eyes, TERD in 4 eyes and persistent VH in 2 eyes. Among 14 eyes that had surgical interventions, poor visual outcomes were observed in 3 eyes due to chronic funnel-shaped retinal detachments, proliferative vitreoretinopathy in 2 eyes and neovascular glaucoma (NVG) in 6 eyes. Table 2 shows the details of RCH management among the study eyes. Secondary surgical procedures were performed in 4 eyes (28.5%). Five patients (27.8%) had cerebellar tumor removal surgeries and 3 patients (16.7%) had nephrectomy surgeries. Tumor regression was achieved in 18 treated eyes (60%). Final retinal attachment was found in 23 eyes (69.7%). NVG developed in 6 eyes (18.2%), and all of these eyes required glaucoma surgeries. Four eyes (12.1%) were complicated with corneal opacities, which required corneal transplantations. Three eyes (9.1%) required eye enucleation. The mean BCVA at last visit was 1.3 ± 1.3 (Snellen = 20/400) and the mean IOP was 16.5 ± 6.4 mmHg. Eleven eyes (33.3%) were blind on the last visit. The change in BCVA was statistically significant (*P* = 0.003). Significantly worse visual outcomes were observed in patients who had lower BCVA on presentation (*P* = 0.002), larger tumor sizes (*P* = 0.040), developed retinal complications (odds ratio 8.143, *P* < 0.001), undergoing retinal surgery (odds ratio 2.615, *P* < 0.001), detached retina on the last follow up (odds ratio 23.734, *P* < 0.001), persistent tumor outcome (odds ratio 6.538, *P* < 0.001), development of NVG (odd ratio 12.172, *P* < 0.001), and corneal complications (odds ratio 7.624, *P* < 0.001). The factors which were associated with worse visual outcomes are summarized in Table 3. None of the treatment modalities was associated with a change in BCVA. The analysis of the effect of various treatments on the change in BCVA is summarized in Table 4.

**Table 1:** The treatment modalities used in eyes with von Hippel Lindau related retinal capillary hemangioblastomas

Treatment	Eyes (*n* = 30)	%
Focal laser photocoagulation	27	90
Cryotherapy	8	26.7
Bevacizumab injection	6	20
Dexamethasone implant	3	10
Aflibercept	1	3.3
Scleral drainage	2	6.7
Pars plana vitrectomy	12	40

**Table 2:** The treatment modalities and their outcomes used in 33 eyes of 18 patients with von Hippel Lindau related retinal capillary hemangioblastoma

Patient /Eye	First treatment	Outcome	Second treatment	Outcome	Third Treatment	Outcome
Patient 1 OD	Focal laser	Regressed lesions	None	N/A	None	N/A
Patient 1 OS	Focal laser	Persistent ERD	PPV + feeder vessel ligation + Endolaser + Cryotherapy	CME	Intravitreal Faricimab	Improved CME
Patient 2 OD	None	N/A	None	N/A	None	N/A
Patient 2 OS	Focal laser	Persistent ERD	PPV EL + cryotherapy	Persistent ERD	None	N/A
Patient 3 OU	Focal laser	Regressed lesions	None	N/A	None	N/A
Patient 4 OS	Focal laser	Persistent ERD	PPV + Cryotherapy	Regressed most lesions except peripherally	Cryotherapy OS for peripheral lesions	All lesions regressed
Patient 5 OD	Focal laser	Persistent activity	Intravitreal Bevacizumab Intravitreal dexamethasone	Persistent ERD	Trans-scleral drainage + Cryotherapy	Recurrent ERD with PVR
Patient 6 OD	Focal laser	Active lesions	Intravitreal Bevacizumab Intravitreal dexamethasone	TERD	PPV + MP + feeder vessel ligation	Chronic funnel shaped TRD
Patient 6 OS	Focal laser	Persistent ERD	PPV + EL + trans-scleral drainage	Persistent ERD	None	N/A
Patient 7 OD	Focal laser	Active lesions	Intravitreal Bevacizumab Intravitreal dexamethasone	TERD	Transscleral drainage + PPV + MP + EL	Chronic funnel shaped TRD and NVG
Patient 7 OS	Focal laser	Regressed lesions	None	N/A	None	N/A
Patient 8 OD	Focal laser	Regressed lesions	None	N/A	None	N/A
Patient 8 OS	Focal laser	Persistent ERD	PPV + EL	Persistent ERD with NVG	None	N/A
Patient 9 OD	focal laser	Persistent ERD	PPV + EL	Persistent ERD with NVG	None	N/A
Patient 9 OS	Focal laser	Regressed lesions	None	N/A	None	N/A
Patient 10 OD	Focal laser	Persistent activity	Intravitreal Aflibercept	Persistent ERD	PPV + cryotherapy	flat retina
Patient 11 OD	Focal Laser	Active lesions	Intravitreal Bevacizumab Intravitreal Dexamethasone	Persistent VH	PPV + feeder vessel ligation + cryotherapy	Regressed lesions and flat retina
Patient 11 OS	Focal Laser	Regressed lesions	None	N/A	None	N/A
Patient 12 OU	Focal Laser	Regressed lesions	None	N/A	None	N/A
Patient 13 OD	PPV + Scleral Buckle	TERD	PPV + feeder vessel ligation + tumor resection + EL	Persistent TERD	PPV + Cryotherapy + SB	Regressed lesions and attached retina with NVG
Patient 13 OS	Focal laser	Active lesions	PPV + Cryotherapy	Regressed lesions	None	N/A
Patient 14 OD	PPV + Cryotherapy + EL + vascular ligation	Recurrent ERD	PPV + feeder vessel ligation + Tumor resection	Recurrent ERD	PPV + MP + retinectomy	Chronic funnel shaped TRD and NVG
Patient 14 OS	Focal laser	Regressed lesions	None	N/A	None	N/A
Patient 15 OD	Focal laser	Regressed lesions	None	N/A	None	N/A
Patient 15 OS	Focal laser	Persistent VH	PPV + feeder vessel ligation + EL	Persistent ERD	PPV + MP + EL	Regressed lesions and flat retina with NVG
Patient 16 OD	None	N/A	None	N/A	None	N/A
Patient 16 OS	Focal laser	Active lesions	Intravitreal Bevacizumab	Regressed lesions	None	N/A
Patient 17 OD	Focal Laser	Active lesions	Intravitreal Bevacizumab	TERD	PPV + MP + EL + SB	Regressed lesions
Patient 17 OS	Cryotherapy	Persistent TERD and PVR	None	N/A	None	N/A
Patient 18 OD	None	N/A	None	N/A	None	N/A
Patient 18 OS	Focal Laser	Regressed lesions	None	N/A	None	N/A

CME: cystoid macular edema; EL: endolaser; MP: membrane peeling, N/A: not applicable; NVG: neovascular glaucoma; OD: right eye; OU: both eyes; OS: left eye; PPV: pars plana vitrectomy; PVR: proliferative vitreoretinopathy; SB: scleral buckle; TERD: tractional exudative retinal detachment, VH: vitreous hemorhage

**Table 3:** The factors which were associated with worse visual outcomes

Factor	Effect on final BCVA (95% CI)	Odds ratio (CI:95%)
Initial BCVA	*P* = 0.002	N/A*
Number of tumors	*P* = 0.408	N/A*
Tumors size	*P* = 0.040	N/A*
Retinal complications	*P* < 0.001	8.143
Retinal surgical interventions	*P* < 0.001	2.615
Detached retina on last visit	*P* < 0.001	23.734
Tumor outcome	*P* < 0.001	6.538
Glaucoma	*P* < 0.001	12.172
Corneal opacities	*P* = 0.016	7.624

BCVA: best-corrected visual acuity; CI: confidence interval; N/A: not applicable

* The odds ratio could not be calculated for factors with numerical variables

**Table 4:** The analysis of the effect of various treatments on the change in BCVA

Treatment	Effect on BCVA (CI:95%)	Odds ratio (CI:95%)
Laser photocoagulation	*P* = 0.845	0.980
Number of laser sessions	*P* = 0.749	N/A*
Number of intravitreal injections	*P* = 0.347	N/A*
Number of surgeries	*P* = 0.176	N/A*
Cryotherapy	*P* = 0.544	0.381
Bevacizumab injection	*P* = 0.740	0.229
Dexamethasone implant	*P* = 0.244	4.062
Scleral drainage	*P* = 0.765	2.639
Vitrectomy	*P* = 0.104	7.727

BCVA: best-corrected visual acuity; CI: confidence interval; N/A: not applicable

* The odds ratio could not be calculated for factors with numerical variables

## Discussion

Although there are numerous papers that described the natural history and genotype-phenotype correlations of RCH in VHL, there are only a handful of papers that describe the treatment outcomes, especially recently, with the increase in variability of treatment modalities. Treatment of RCH can be challenging because of the presence of bilateral multiple tumors and the occurrence of new tumors [7]. Various treatment modalities, including observation, laser photocoagulation, cryotherapy, plaque radiotherapy, and vitreoretinal surgery, have been described [7] .

Among 7 patients who were confirmed to have pathogenic variants in VHL, one patient had complete deletions of exon 3. This patient had unilateral RCH with macular edema and exudation and was treated with multiple laser photocoagulations with stable vision at the level of counting fingers.

The mean size of RCH in this cohort was 1.5 ± 0.9 DD. Krizovic et al. reported the outcomes of laser photocoagulation in RCH with a median size of 0.25 DD, which achieved tumor inactivation in 100% of tumors < 1DD [13]. Krzystolic et al. reported the outcome of surgical interventions in patients with RCH, and the average tumor size was 2 DD [14]. These findings indicate that the clinical presentation of RCH might be more severe in larger tumors. The variability of treatment modalities used in our patients who had an in-between average tumor size further supports this observation. Another challenge which was faced in this cohort was the variability of tumor size within the same eyes, which resulted in the need for multiple laser sessions or cryotherapy to control the activity of all detected tumors.

**Fig. 3:**
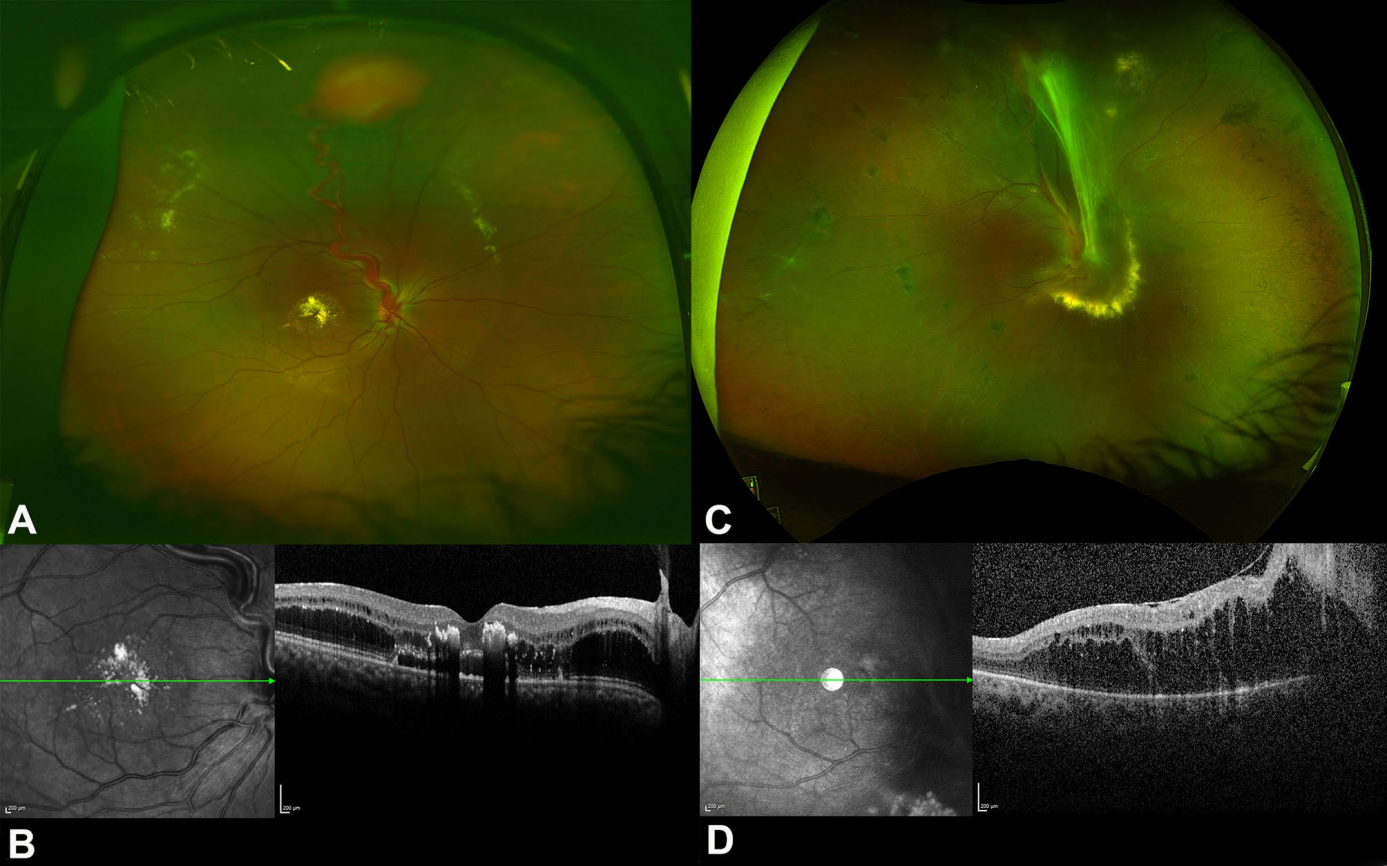
Progressive tractional retinal detachment (TRD) after the application of local treatment for retinal capillary hemangioblastoma in a patient with von Hippel-Lindau (VHL) disease. A is a color fundus photo of the right eye showing a superior RCH with prominent feeder blood vessels and macular exudations, and thickening. B is a spectral domain optical coherence tomography (SD-OCT) of the right eye showing fluid accumulation in the outer retinal layers and retinal exudations. C is a color fundus photo of the same eye after two sessions of laser photocoagulation, showing regression of RCH and the feeding blood vessels, resulting in tractional band formation extending to the macula and laser scars. D is an SD-OCT showing a thick epiretinal membrane exerting macular traction and separation of the retinal layers

The differences in the sizes of the tumors and the presence of large RCH in the study eyes resulted in higher risks of activity and subretinal fluid accumulation in 83.3% of eyes included in this study, necessitating treatment. Similar observations were found among eyes that had laser photocoagulation, as subretinal or intraretinal fluids were observed in 21 eyes, which had a median tumor size of 2 DD [13]. The larger tumors in this cohort required more laser sessions on average (1.96 vs. 1.3 laser sessions) [13]. Previous studies have shown that the success of laser photocoagulation in RCH can reach up to 100% [7, 13, 15]. This was not observed in our study, as only 37% of RCH were controlled with laser alone. In addition, progressive retinal traction and detachment can be observed after laser photocoagulation due to the prominence of the regressed feeder blood vessels (Fig. 3A-D).

Combined laser photocoagulation is considered a second line of treatment in large tumors > 3 DD [13, 16, 17]. Among our cohort, 5 out of 8 patients (62.5%) who were treated with laser photocoagulation required retinal surgical interventions. These findings indicate that primary surgical interventions are considered a reasonable approach in these tumors [14].

The most common cause of retinal surgical interventions was persistent ERD, followed by TERD. Although a combination of both components frequently leads to retinal detachment in RCH [14], the prominence of ERD was noted in 7 eyes and TRD in 4 eyes, which were not treated with prior laser.

Among 14 eyes that had surgical interventions, poor visual outcomes were observed in 3 eyes due to chronic funnel-shaped retinal detachments, proliferative vitreoretinopathy in 2 eyes and neovascular glaucoma (NVG) in 4 eyes. On the other hand, the 1-month outcomes after surgical interventions were more favorable [14]. These findings highlight the importance of long-term follow-up in these patients after retinal surgeries.

The rate of blindness in this cohort was 33.3% higher than the previously reported 8.2%. The visual outcomes in our study were significantly impacted by the development of ocular complications, especially if surgical interventions were warranted to manage these complications. Recently, a consensus guidelines reported that the impression of VHL-related RCH retinal complications are common with or without treatment, and these complications are known to affect visual outcomes [18]. Recently, belzutifan, a hypoxia-inducible factor 2a (HIF-2a), was approved for the management of extraocular manifestations of VHL disease [19]. The results of a single-arm phase II study found beneficial outcomes of belzutifan in controlling the activity of RCH [20].

One patient with a history of consanguinity manifested VHL and ESCS syndromes. Notably, her left eye, which manifested multiple RCH, was the only eye in this cohort to manifest CNVM. To our knowledge, CNVM has not been reported in VHL disease. On the other hand, CNVM was found to be one of the features of ESCS [21]. In addition, her contralateral eye, which did not manifest RCH, had multiple cystic changes at multiple retinal levels. Although cystoid macular edema has been reported in VHL disease and was found in one of the study eyes (Fig. 3A) [22, 23], the fluid accumulation is typically more prominent in the outer retina and is accompanied by retinal exudations. In addition, the formation of cystoid retinal spaces is a well-known feature of ESCS, which can eventually progress to cause full-thickness macular hole formation [24]. In addition, there was no leakage of fluorescein in the macular area on the FFA, which also rules out CME. While clinical features can be distinctive in differentiating between VHL and ESCS features, the ERG might be less helpful. Patients with VHL with or without RCH were found to have delayed implicit times of b waves and maximal a waves, indicating dysfunctional photoreceptors and inner retinal cells [11]. We are not aware of any previous co-occurrence of VHL and retinal dystrophy. This might be due to the frequent consanguinity in the Saudi population.

Our study has several limitations. First, genetic confirmation was not performed in all patients and, the diagnosis was established on the basis of systemic findings and the typical ocular features of RCH. Second, because of the rarity of the disease, only retrospective methodology could be used to conduct this study. The study eyes could not be categorized on the basis of tumor size or location due to the findings of multiple lesions of different sizes and locations per eye. In addition, there was no standardization of the number or the order of treatment modalities used due to the difference in clinical judgment by treating physicians. Another limitation was the difficulty in evaluating the effect of each treatment modality separately, because of the heterogeneity of the clinical pictures presented to the treating physicians.

In conclusion, the management outcomes in VHL-related RCH are variable and depend on the lesion size and location, severity of the clinical picture on presentation and the development of complications. Guarded visual outcomes are not uncommon in these patients, especially if advanced ocular complications developed, surgical interventions were warranted. Encountering atypical clinical features in these patients might suggest exploring further diagnostic possibilities.

## Supplementary Information

Below is the link to the electronic supplementary material.

Supplementary Material 1

## Data Availability

No datasets were generated or analysed during the current study.

## Abbreviations

BCVA: Best-corrected visual acuity
ESCS: Enhanced S-cone syndrome
IOP: Intraocular pressure
KKESH: King Khaled Eye Specialist Hospital
RCH: Retinal capillary hemangioblastoma
TRD: tractional retinal detachment
T-ERD: Tractional exudative retinal detachment
VEGF: Vascular endothelial growth factor
VHL: Von Hippel Lindau
