# Supplementary material for: The clinical features and real world treatment outcomes in von Hippel Lindau related retinal capillary hemangioblastomas
Source: Int J Retina Vitreous. 2025 Aug 18;11:93. doi: 10.1186/s40942-025-00721-1 (PMC12359959; doi:10.1186/s40942-025-00721-1)
Supplement: Supplementary file 1 — Supplementary Material 1 [file 40942_2025_721_MOESM1_ESM.docx]

Table 1: The results of whole exome sequencing in 7 patients with von Hippel Lindau disease

| Variant | Effect on Protein | Gene | Zygosity |
| --- | --- | --- | --- |
| c.481C>T | (p.Arg161*) | *VHL* | Heterozygous |
| c.227_229delTCT | (p.phe76del) | *VHL* | Heterozygous |
| c.390dupT | (p.Asn131*) | *VHL* | Heterozygous |
| c.486C>G | p.Cys162trp) | *VHL* | Heterozygous |
| c.256C>T | (p.Pro86ser) | *VHL* | Heterozygous |
| deletion of exon 3 | deletion of exon 3 | *VHL* | Heterozygous |
| c.452T>C | (p.Ile151Thr) | *VHL* | Heterozygous |
| c.932 G>A | (p.Arg311Gln) | *NR2E3* | Homozygous |
